# Immunization with desmoglein 3 induces non-pathogenic autoantibodies in mice

**DOI:** 10.1371/journal.pone.0259586

**Published:** 2021-11-03

**Authors:** Katharina Boch, Sören Dräger, Detlef Zillikens, Christoph Hudemann, Christoph M. Hammers, Sabrina Patzelt, Enno Schmidt, Ewan A. Langan, Rüdiger Eming, Ralf J. Ludwig, Katja Bieber

**Affiliations:** 1 Department of Dermatology, University of Lübeck, Lübeck, Germany; 2 Department of Dermatology and Allergology, Phillips-Universität Marburg, Marburg, Germany; 3 Lübeck Institute for Experimental Dermatology, University of Lübeck, Lübeck, Germany; 4 Dermatological Sciences, University of Manchester, Manchester, United Kingdom; University of Texas at San Antonio, UNITED STATES

## Abstract

**Background:**

Pemphigus vulgaris (PV) is a rare autoimmune blistering disease characterized by the development of autoantibodies targeting desmoglein (Dsg) 3, but also against Dsg1 in mucocutaneous disease. Given that existing PV animal models only recapitulate aspects of the disease, we aimed to establish a more comprehensive disease model based on the immunization of mice with PV autoantigen(s).

**Methods:**

The following immunization strategies were tested: (i) C57Bl/6J, B6.SJL-H2s C3c/1CyJ, DBA2/J, or SJL/J mice were immunized with recombinant murine Dsg3 (mDsg3), (ii) DBA2/J and SJL/J mice were immunized with mDsg3 and additionally injected a single non-blister inducing dose of exfoliative toxin A (ETA), and (iii) DBA2/J and SJL/J mice were immunized with human Dsg (hDsg) 1 and 3.

**Results:**

Despite the induction of autoantibodies in each immunization protocol, the mice did not develop a clinical phenotype. Tissue-bound autoantibodies were not detected in the skin or mucosa. Circulating autoantibodies did not bind to the native antigen in indirect immunofluorescence microscopy using monkey esophagus as a substrate.

**Conclusion:**

Immunization with PV autoantigens induced non-pathogenic Dsg1/3 antibodies, but did not cause skin/mucous membrane disease in mice. These findings, confirmed by failure of binding of the induced autoantibodies to their target in the skin, suggest that the autoantibodies which were formed were unable to bind to the conformational epitope present *in vivo*.

## Introduction

Pemphigus vulgaris (PV) is a rare autoimmune blistering disease affecting the skin and mucous membranes. The disease is characterized by the production of autoantibodies targeting desmoglein (Dsg) 3, but also against Dsg1 in the mucocutaneous disease variant [1, 2]. The pathogenesis of PV can be divided into two phases: (i) loss of tolerance with subsequent autoantibody production (priming phase) and (ii) binding of autoantibodies and subsequent intraepidermal acantholysis (effector phase). PV is a well-characterized, prototypical human autoimmune disease. Establishment of an animal model would not only shed light on disease pathogenesis, but it may also provide insight into a range of autoimmune diseases. However, a pemphigus mouse model duplicating all aspects of the human pathogenesis is currently lacking. Pre-clinical pemphigus models have largely been based on passive transfer of autoantibodies into immunocompetent neonatal mice [3] or the adoptive transfer of Dsg3-specific lymphocytes into immunodeficient mice [4]. PV autoantibodies can also be induced by immunization in a humanized mouse model, although the mice do not develop clinical manifestations of PV [5]. While active disease models for epidermolysis bullosa acquisita (EBA) [6] and bullous pemphigoid (BP) [7] have been successfully established, the set-up of an immunization-based model for PV has been more challenging, possibly indicating differences in the loss of tolerance in these autoimmune skin conditions. Immunization of C57BL/6N, BALB/c and C3H/HeJ mice with recombinant murine Dsg3 (mDsg3) emulsified in Freund’s complete adjuvant (CFA)/incomplete Freud`s adjuvant (IFA) led to the production of anti-Dsg3 antibodies which did not recognize native mouse Dsg3 and did not induce skin/mucous membrane lesions [4]. We aimed to overcome these shortcomings by using TiterMax^™^ as adjuvant and mice known to be highly susceptible to developing autoantibody-mediated diseases.

## Material and methods

### Animals

C57Bl/6J, B6.SJL-H2s C3c/1CyJ, DBA2/J or SJL/J mice at a similar sex distribution were obtained from Charles River Laboratories (Wilmington, Massachusetts, USA), Janvier Labs (Le Genest-Saint-Isle, France) or from in-house breeding. Mice aged 8–10 weeks were used for the experiments. Mice were kept in a conventional animal house in filter top cages (≤5 mice/cage, 22°C) with a 12h light/dark cycle and with *ad libitum* access to water and standard pelleted food. All immunizations, blood sampling and endoscopy of the oral cavity were performed under ketamine/xylazine anesthesia, using i.p. administration of a mixture of ketamine (100 mg/g, Sigma-Aldrich) and xylazine (15 mg/g, Sigma-Aldrich). Mice were anaesthetized by ketamine/xylazine anesthesia and sacrificed by cervical dislocation. Experiments were refined to minimize suffering of experimental animals during all procedures (S3 Table). The protocol was approved by the Committee on the Ethics of Animal Experiments of Schleswig-Holstein (Protocol Number: 62-5/17) prior to beginning the research. All animal experiments were carried out in strict accordance with the recommendations in the Guide for the Care and Use of Laboratory Animals of the National Institutes of Health. Research staff received appropriate training in animal care.

### Recombinant protein expression

Murine desmoglein 3 (mDsg3) and human desmoglein 1/3 (hDsg1/hDsg3) expression and purification was performed using a His-tag, as previously described [8, 9].

### Immunization protocol

All subcutaneous (s.c.) immunizations were carried out with 100 μg mDsg3 or a combination of 30 μg hDsg1 and 70 μg hDsg3 emulsified in the adjuvant TiterMax^™^ (Sigma-Aldrich, St. Louis, Missouri, USA). Immunizations occurred at day 0, 14, 35, and 56, adapted from a mouse model of immunization-induced EBA [6]. Subsequent injections were given (in this order): in both foot pads, in the tail base, in the right lateral abdominal region and in the left lateral abdominal region. The follow-up was 84 days. Mice were weighed weekly and clinically examined for evidence of cutaneous/mucosal lesions. Serum samples were obtained every second week. A high-resolution mouse endoscope (HOPKINS Optik 64019BA; Karl StorzAidaVet, Tuttlingen, Germany) was used to determine the extent of oral lesions as described [10]. Biopsies of the skin, buccal mucosa, and esophagus were obtained at the end of the observation period.

### Exfoliative toxin A

Exfoliative toxin A (ETA) dose-finding experiments were performed in DBA2/J and SJL/J mice. The following dosages were used: 0.5/1.0 μg ETA/g mouse. Of these, 0.5 μg ETA/g mouse was the highest dose that did not induce skin blistering within 48 hours when injected intraperitoneally (i.p.) into mice (S1 Table). ETA (Toxin Technology, Inc., Sarasota, Florida, USA) i.p injected with 0.5 μg/g body weight (bw) on day 28.

### Direct and indirect immunofluorescence (IF) microscopy

Tissue-bound IgG was detected by direct IF microscopy (DIF) in perilesional biopsies using fluorescein isothiocyanate (FITC)-conjugated donkey anti-mouse IgG (1:100; Jackson Immuno Research Europe, Suffolk, UK). Binding of circulating anti-Dsg3 antibodies to monkey esophagus was tested by indirect IF (IIF) using FITC-labeled donkey anti-mouse IgG as detection antibody (1:100; Jackson Immuno Research Europe, Suffolk, UK); samples of mouse sera were diluted 1:100. All IF stainings were evaluated using a Keyence BZ-9000 microscope (Keyence, Neu-Isenburg, Germany). Immunoreactivity was quantified with ImageJ software (National Institutes of Health, Bethesda, MD, USA).

### Enzyme-linked immunosorbent assay (ELISA)

Serum levels of circulating anti-mDsg3 antibodies were quantified by ELISA using mouse IgG quantification sets following manufacturer`s protocol (Bethyl Laboratories, Inc., Montgomery, Tx, USA) with modifications for plate coating. In detail, each well was coated with 500 ng recombinant mDsg3 in coating buffer (0.05 M Na_2_CO_3_). After blocking with 1% bovine serum albumin (BSA) in phosphate buffered saline plus Tween^®^ 20 detergent (PBST), series of diluted samples were added and incubated for 60 min. Bound Abs were detected by HRP-conjugated goat anti-mouse Abs of the respective subclass (Bethyl Laboratories) and tetramethylbenzidine (Invitrogen, Carlsbad, CA, USA). The enzymatic color reaction was stopped by 2 M sulfuric acid, and the change in OD was measured with a GlowMax-Multi Detection System (Promega Corporation, Madison, Wisconsin, USA) at 450 nm. Standard reference curves were established by using the provided mouse reference sera (Bethyl Laboratories). Anti-hDsg1/3 antibodies of the mice immunized with hDsg were quantified following manufacturer`s protocol (Euroimmun AG, Lübeck, Germany) and were modified by using an anti-mouse IgG HRP-conjugated secondary antibody (Bethyl Laboratories). To exclude the immunogenic effects of the His-tag, pre-adsorption was performed by addition of 3 μl his-tagged murine collagen (His-mCOL)7 before serum sample dilution. Antibody binding was determined at the absorbance of 450 nm.

### Detection of mDsg3-reactive B/plasma cells in draining lymph nodes

For immunofluorescence double staining of mDsg-specific B and plasma cells, recombinant mDsg3 was labelled with Atto 488 (Atto 488 Protein Labeling Kit, Merck Millipore, Burlington, MA, USA) following the manufacturer´s protocol. Cryosections of murine draining popliteal lymph nodes (12 weeks after immunization with mDsg3) were fixed, blocked with 1% BSA and double stained with mDsg3-Atto488 (1:5) and biotinylated anti-B220/CD45R (clone RA3-6B2, Novus Biologicals, Littleton, CO, USA) for 1h. After washing, Streptavidin-DyLight594 (Dianova, Hamburg, Germany) was used for detection of B220 and sections were counterstained with DAPI. A similar protocol had been used previously to detect autoantigen-specific B cells in experimental epidermolysis bullosa acquisita [11, 12].

## Results and discussion

**Fig 1:**
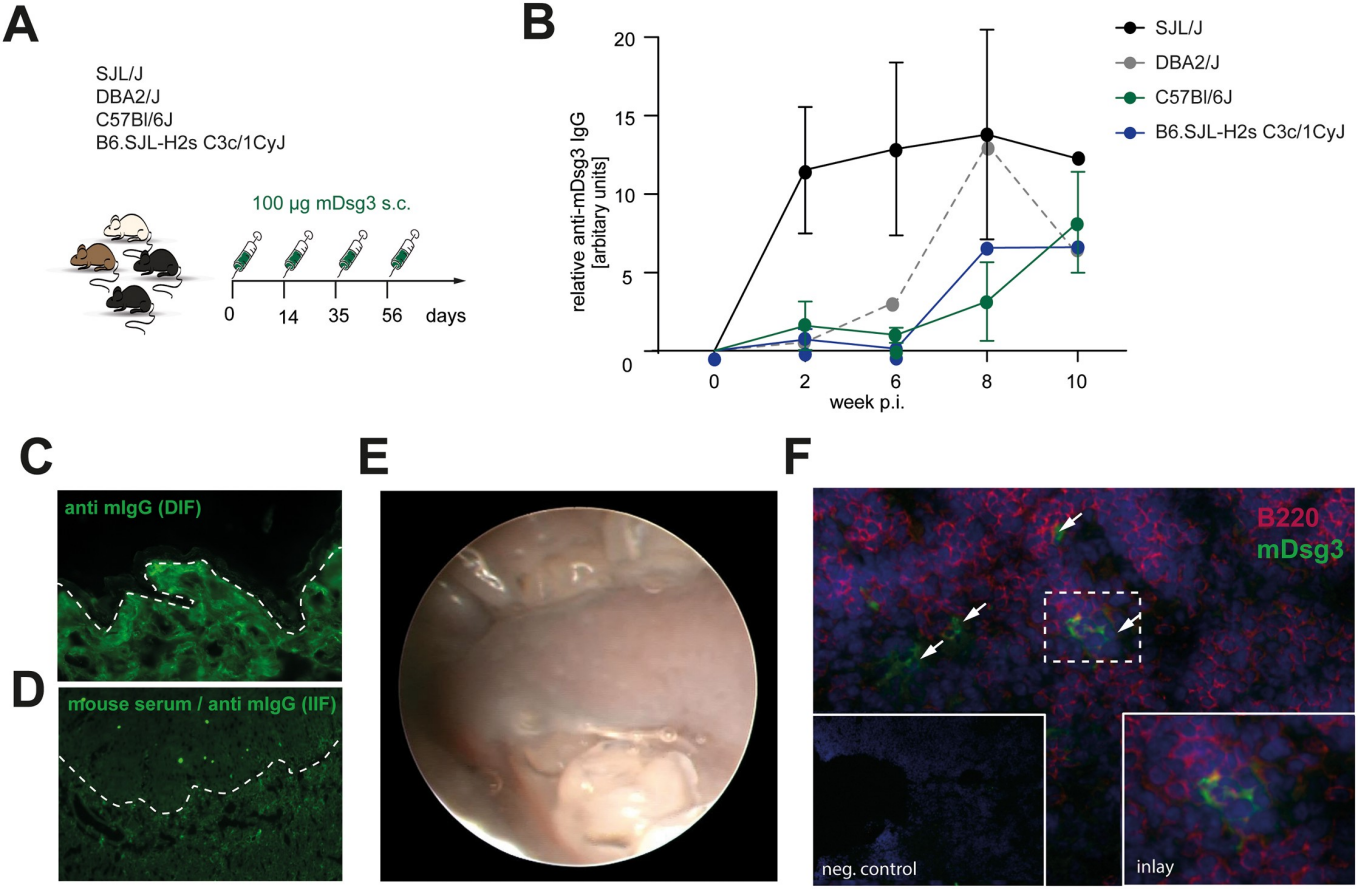
Immunization with murine desmoglein 3 induces antibodies against Dsg3 but no clinical PV phenotype. (A) C57Bl/6J, B6.SJL-H2s, DBA2/J or SJL/J mice were immunized 4x with mDsg3 emulsified in TiterMax^™^ on days 0, 14, 35, 56. (B) Dsg3-specific IgG autoantibody titers of C57Bl/6J (n = 4), B6.SJL-H2s (n = 4), DBA2/J (n = 4) and SJL/J (n = 4) mice immunized 4x with mDsg3 emulsified in TiterMax^™^ on days 0, 14, 35, and 56. (C) Direct immunofluorescent staining for anti-mouse-IgG (facial skin) at day 84. (D) Monkey esophagus was incubated with serum from immunized mice (day 84) and stained for anti-mouse-IgG. In both direct and indirect IF microscopy, no binding of IgG to the epidermis was detected. Dotted line: Dermal-epidermal junction. (E) Representative picture of endoscopy of the oral cavity showed no mucous membrane lesions at day 84. (F) The formation of mDsg3-specific B or plasma cells was confirmed by immunofluorescence of draining lymph nodes (day 84). B220: red, recombinant mDsg3 protein: green, arrows indicate Dsg3-specific B cells.

Immunization of C57Bl/6J (*n = 8*), B6.SJL-H2s C3c/1CyJ (*n = 8*), DBA2/J (*n = 8*) and SJL/J (*n = 8*) mice with mDsg3 (Fig 1A) led to the induction of antigen-specific IgG in each mouse strain. Serum levels of circulating anti-mDsg3 antibodies were quantified by ELISA (Fig 1B). Serum concentrations of anti-mDsg3 antibodies were the highest in SJL/J mice and peaked at week 2. In contrast, the C57Bl/6J B6.SJL-H2s C3c/1CyJ and DBA2/J mouse strains seemed to require a repeated antigen exposure in order to reach peak autoantigen production. Anti-Dsg3 antibodies of DBA2/J mice reached peak in week 6, showing the highest levels of autoantibodies in week 8. In comparison C57Bl/6J and B6.SJL-H2sC3 mice showed lower titers of anti-Dsg3 antibody levels. Tissue-bound anti-mouse Dsg3 IgG antibodies were neither detected in skin (Fig 1C) nor mucosal biopsies (S2 Table). Furthermore, anti-mouse Dsg3 IgG antibodies from the serum of immunized mice did not bind to the native antigen in indirect immunofluorescence microscopy using monkey esophagus as a substrate (Fig 1D). There was no evidence of skin/mucous membrane lesions in the mice (Fig 1E). Immunohistochemistry staining of murine lymph nodes detected Dsg3-reactive B-cells, suggesting that a specific immune response to the immunization with the PV antigen (Fig 1F). Hence, immunization of autoimmune-prone mouse strains (C57Bl/6J, B6.SJL-H2s C3c/1CyJ, DBA2/J, SJL/J) with PV autoantigens led to formation of autoantibody-specific B cells and non-pathogenic Dsg3 autoantibodies. In line with our results, autoantibodies against Dsg3 have been detected in mDsg3 immunized C57BL/6N, BALB/c and C3H/HeJ mice, but failed to bind to the native form of murine Dsg3 [4].

**Fig 2:**
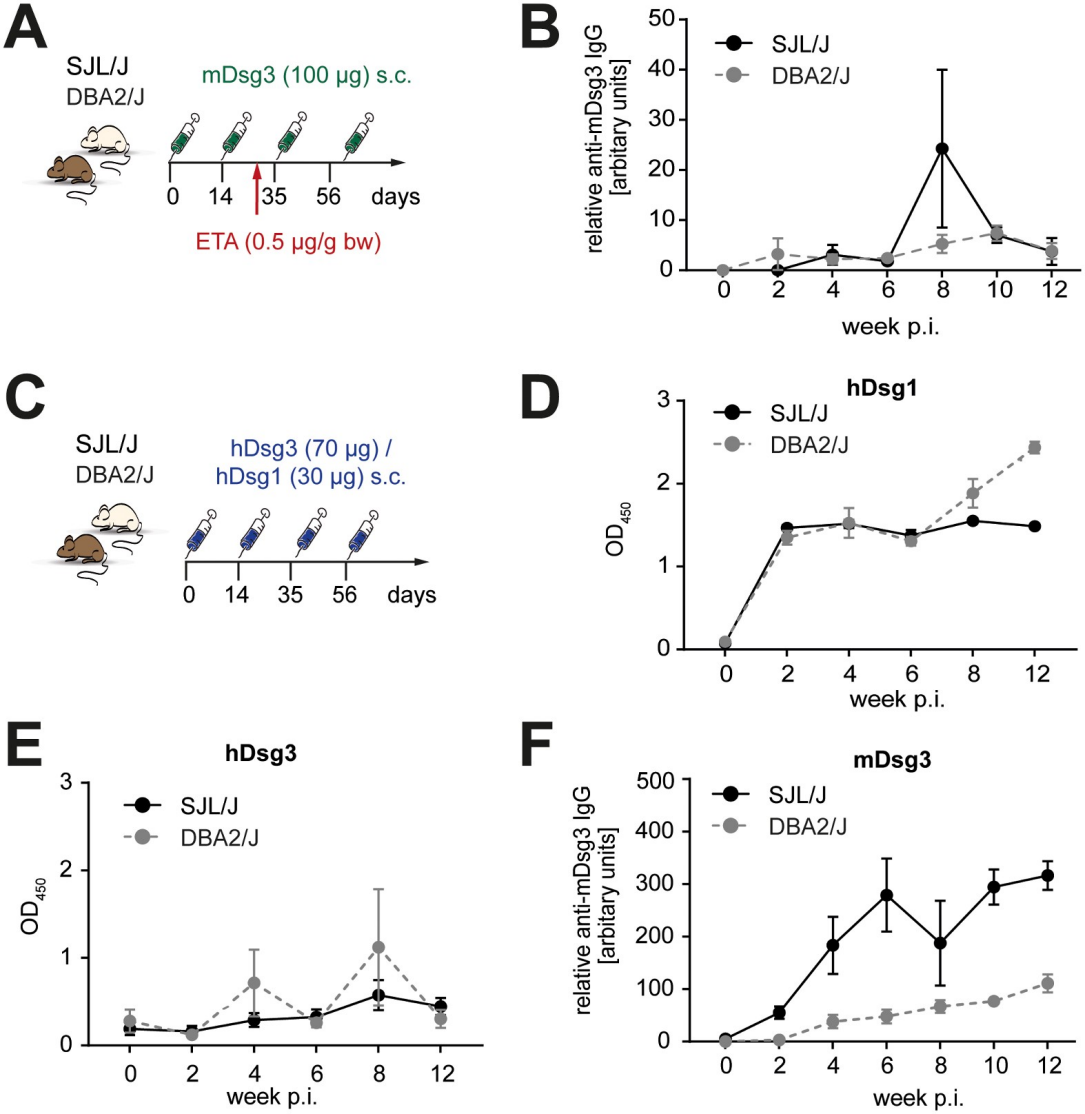
Immunization with murine desmoglein 3 and additional ETA or human desmoglein induce antibodies against Dsg3, but no clinical PV phenotype. (A) Modified 4x immunization protocol (additional injection of 0.5 μg/ml ETA at day 28) was tested in DBA2/J and SJL/J mice on day 0, 14, 35, 56. The s.c. immunization was carried out with 100 μg mDsg3 emulsified in TiterMax^™^. (B) Dsg3-specific IgG autoantibody titers of DBA2/J mice (n = 4) and SJL/J mice (n = 3). (C) Modified 4x immunization protocol was tested in DBA2/J and SJL/J mice on day 0, 14, 35, 56. The s.c. immunization was carried out with 30 μg human (h) Dsg1 and 70 μg hDsg3 emulsified in TiterMax^™^ (C). IgG autoantibody titers of DBA2/J mice (n = 4) and SJL/J (n = 5) were analyzed for their activity against (D) hDsg1, (E) hDsg3 and (F) cross-reactivity with mDsg3.

The failure of immunization with mDsg3 to induce pathogenic Dsg3 autoantibodies may have been explained by the inability of the induced anti-Dsg3 IgG to bind to the target antigen *in vivo*. To address this experimentally, we repeated the experiments with the addition of ETA, a serine protease hydrolysing a single peptide bond (GLu381-Gly382) located between EC3 and EC4 of Dsg1 [13], which results in the development of superficial cutaneous blistering. In fact, even at non-blister-inducing concentrations, ETA weakens the adhesion between keratinocytes and thus may enable a better binding of Dsg3 autoantibodies to their target antigen [14]. Based on the obtained results from the first set of experiments and to reflect the 3R`s of animal research [15], the modified immunization protocol was restricted to DBA2/J and SJL/J mice. DBA2/J *(n = 4*) and SJL/J (*n = 4*) mice were immunized with mDsg3 and additionally treated with a non-blister inducing dose of ETA (Fig 2A). Again, although a robust anti-mouse Dsg3 immune response was induced (Fig 2B), these autoantibodies did not bind to their target antigen *in vivo* as evidenced by absence of IgG deposition in the skin of immunized mice obtained at week 12 after the first immunization. After addition of ETA to the immunization protocol, a pemphigus-related clinical phenotype was not observed. As shown in our preceding experiments, anti-Dsg3 antibodies did not bind to the autoantigen *in vivo*. This suggests that the reciprocal compensation of Dsg1/3 was not altered by the Dsg1 disrupting effect of ETA.

In general, the development of experimental models of autoimmune diseases is a challenge due to the ability of the immune system to develop tolerance to autoantigens. In order to induce autoimmunity *in vivo*, self-tolerance mechanisms must be overcome. Hence it is difficult to stimulate the immune system by the application of homologous antigens and to induce the formation of autoantibodies. The use of xenogeneic antigens can promote the loss of tolerance towards autoantigens. By injecting the human PV antigens into mice, we aimed to induce a human/murine cross-reaction and the break of immune tolerance. This had already proved successful in autoimmune animal model used to study rheumatoid arthritis [16, 17]. Furthermore, immunization of humanized HLA-DR4 transgenic mice with human Dsg3 led to the production of anti-Dsg3 antibodies which did not bind to mouse skin but induced the loss of cell-cell adhesion ex *vivo* [5]. Thus, in subsequent experiments, DBA2/J (n = 4) and SJL/J (n = 6) mice were immunized with hDsg1/3 (Fig 2C). These experiments were also seen as a potential alternative strategy because the homology of mDsg3 and hDsg3 is as high as 85.6% [18, 19]. In line with these findings, VH1‑46 antibody heavy chain gene usage by Dsg3-reactive B cells is commonly observed in PV [20] and, interestingly, the pathogenic mouse monoclonal anti-Dsg3 antibody (AK23) [14] is transcribed from a gene that is highly homologous to the human VH1‑46 gene, pointing to the similarity in response to Dsg3 in humans and mice. As in the previous experiments, despite the presence of circulating anti-human Dsg1/3–reactive IgG (Fig 2D and 2E), the mice did not develop PV-like skin changes (S2 Table). Similar to the previous results, these autoantibodies did not bind to Dsg-expressing tissues (mouse skin, mouse mucous membranes, monkey esophagus) by direct or indirect IF microscopy (S2 Table). Human Dsg1/3-immunized mice showed IgG cross-reactivity with mouse Dsg3 (Fig 2F).

In addition to B cells, there is evidence that supports a role for CD4+ T cells in Dsg-autoantibody induction [21–23]. Autoreactive CD4+ T cells regulate the autoantibody production by interacting with autoreactive B-cells. Some HLA class II haplotypes that have been associated with PV seem to encode MHC that are particularly suitable to present specific Dsg3 peptides to CD4+ T cells [24, 25]. The mouse strains used herein cover a wide range of MHC haplotypes (H2b: C57Bl/6J, C57BL/6N / H2d: BABL/c / H2k: C3H/HeJ / H2s: SJL/J, B6.SJL-H2s C3c/1CyJ / H2q: DBA2/J). Thus, the lack of disease-causing autoantibodies following immunization may be also due to the presentation of Dsg3 on these particular MHC molecules that presumably does not include pathogenically relevant epitopes. Therefore, to induce pathogenic anti-Dsg3 antibodies, mice may be immunized with certain proteins located within recombinant Dsg rather than the entire molecule, enhancing the autoantigen/HLA interaction by a selective peptides/protein HLA interaction and leading to specific stimulation of autoreactive CD4+ T cells, which in turn triggers Dsg-specific B cells to generate autoantibodies to that distinct antigen. Another possibility for the lack of blister-inducing Dsg3 autoantibodies may be the choice of adjuvant (TiterMax^™^) [4]. However, we believe that the latter is not the case, as TiterMax^™^ has been been employed to induce experimental EBA [6] and BP [7].

## Conclusion

Our results suggest that anti-Dsg IgG antibodies induced by the different immunization strategies were not able to bind to the conformational epitope present *in vivo* but rather to non-conformational residues of the antigen. This underscores the view that autoimmune disease develops in a multi-step fashion that includes not only the formation of pathogenic autoantibodies but also the recognition of the appropriate epitope to subsequently induce tissue pathology.

## Supporting information

S1 Table Dose escalation study of exfoliative toxin A. Dosage finding of exfoliative toxin A (ETA) and the correlating skin lesion. The dose escalation study was performed stepwise. Abbreviation: ETA, Exfoliative toxin A, g, gram, μg, microgram.
(DOCX)

S2 Table Result database. Mice were weekly weighed and clinically examined for evidence of cutaneous/mucosal lesions to determine primary endpoints. Secondary endpoints were defined by direct immunofluorescence microscopy (DIF) of perilesional biopsies and indirect immunofluorescence microscopy (IIF) (semi-quantitative evaluation of circulating autoantibodies) as well as enzyme-linked immunosorbent assay (ELISA, quantitative evaluation of circulating autoantibodies) of serum samples. At the end of the observation period an oral endoscopy was performed to determine the oral mucosa involvement. Immune staining was performed to detect autoantigen-specific B cells (mDsg3-reactive B/plasma cells) in draining lymph nodes. An adverse events score was performed to sum up all side adverse effects. **Abbreviation**: DIF, direct immunofluorescence microscopy; ELISA, Enzyme-linked immunosorbent assay; ETA, Exfoliative toxin A; hDg1/3, human Desmoglein 1 and 3; IIF, indirect immunofluorescence microscopy; mDsg3, murine Desmoglein 3; n.d., not done; SD, standard deviation.
(DOCX)

S3 Table Adverse events score. The sum of all side adverse effects was added to a total sum.
(DOCX)

S4 Table Results adverse event score. The mean and standard deviation (SD) of the adverse effects of the experiments is summarized in here. Immunizations did not increase the burden of mice during as the adverse events score is not significantly changed.
(DOCX)
