# Supplementary material for: Immunization with desmoglein 3 induces non-pathogenic autoantibodies in mice
Source: PLoS One. 2021 Nov 3;16(11):e0259586. doi: 10.1371/journal.pone.0259586 (PMC8565724; doi:10.1371/journal.pone.0259586)
Supplement: S1 Table — Dosage finding of exfoliative toxin A (ETA) and the correlating skin lesion. The dose escalation study was performed stepwise. Abbreviation: ETA, Exfoliative toxin A, g, gram, μg, microgram. (DOCX) [file pone.0259586.s001.docx]

| **Mouse ID** | **ETA dosage** | **Skin lesions** |
| --- | --- | --- |
| SJL/J  #1 | 0.5 µg ETA/g mouse | No skin lesions |
| SJL/J  #2 | 0.5 µg ETA/g mouse | No skin lesions |
| SJL/J  #3 | 0.5 µg ETA/g mouse | No skin lesions |
| DBA2/J  #1 | 0.5 µg ETA/g mouse | No skin lesions |
| DBA2/J  #2 | 0.5 µg ETA/g mouse | No skin lesions |
| DBA2/J  #3 | 0.5 µg ETA/g mouse | No skin lesions |
| DBA2/J  #1 | 1 µg ETA/g mouse | Hair loss, erosion, initial blistering |
| DBA2/J  #2 | 1 µg ETA/g mouse | Hair loss, erosion, initial blistering |
| SJL/J  #3 | 1 µg ETA/g mouse | Hair loss, erosion, initial blistering |

**S1 Table. Dose escalation study of exfoliative toxin A.**

Dosage finding of exfoliative toxin A (ETA) and the correlating skin lesion. The dose escalation study was performed stepwise.

Abbreviation: ETA, Exfoliative toxin A, g, gram, µg, microgram
