# Supplementary material for: Immunization with desmoglein 3 induces non-pathogenic autoantibodies in mice
Source: PLoS One. 2021 Nov 3;16(11):e0259586. doi: 10.1371/journal.pone.0259586 (PMC8565724; doi:10.1371/journal.pone.0259586)
Supplement: S2 Table — Mice were weekly weighed and clinically examined for evidence of cutaneous/mucosal lesions to determine primary endpoints. Secondary endpoints were defined by direct immunofluorescence microscopy (DIF) of perilesional biopsies and indirect immunofluorescence microscopy (IIF) (semi-quantitative evaluation of circulating autoantibodies) as well as enzyme-linked immunosorbent assay (ELISA, quantitative evaluation of circulating autoantibodies) of serum samples. At the end of the observation period an oral endoscopy was performed to determine the oral mucosa involvement. Immune staining was performed to detect autoantigen-specific B cells (mDsg3-reactive B/plasma cells) in draining lymph nodes. An adverse events score was performed to sum up all side adverse effects. Abbreviation: DIF, direct immunofluorescence microscopy; ELISA, Enzyme-linked immunosorbent assay; ETA, Exfoliative toxin A; hDg1/3, human Desmoglein 1 and 3; IIF, indirect immunofluorescence microscopy; mDsg3, murine Desmoglein 3; n.d., not done; SD, standard deviation. (DOCX) [file pone.0259586.s002.docx]

| Mouse ID | Weight loss  -/+ | Skin lesions  (erosions/crusts/alopecia)  -/+ | Mucosal lesions  (erosions/blisters)  -/+ | DIF  +/- | IIF  +/- | ELISA  +/- | mDsg3-reactive B/plasma-cells  +/- | Endpoint  +/- | Adverse events score  (mean ± SD) |
| --- | --- | --- | --- | --- | --- | --- | --- | --- | --- |
| C57Bl/6J  #1 | - | - | n.d | Skin: -  Mucosa: - | - | + | n.d. | - | 0 (0-1) |
| C57Bl/6J  #2 | - | - | - | Skin: -  Mucosa: - | - | + | + | + | 1 (0-1) |
| C57Bl/6J  #3 | - | - | - | Skin: -  Mucosa: - | - | + | + | + | 1 (0-1) |
| C57Bl/6J  #4 | - | - | n.d. | Skin: -  Mucosa: - | - | + | + | - | 0 (0-2) |
| C57Bl/6J  #5 | - | - | - | Skin: -  Mucosa: - | - | + | + | + | 1 (0-1) |
| C57Bl/6J  #6 | - | - | n.d. | Skin: -  Mucosa: - | - | + | + | - | 0 (0-2) |
| C57Bl/6J  #7 | - | - | n.d. | Skin: -  Mucosa: - | - | + | + | - | 0 (0-1) |
| C57Bl/6J  #8 | - | - | - | Skin: -  Mucosa: - | - | + | + | + | 0 (0-3) |
| B6.SJL-H2s C3c/1CyJ  #1 | - | - | n.d. | Skin: -  Mucosa: - | - | + | + | - | 0 (0-1) |
| B6.SJL-H2s C3c/1CyJ  #2 | - | - | - | Skin: -  Mucosa: - | - | + | + | + | 1 (0-2) |
| B6.SJL-H2s C3c/1CyJ  #3 | - | - | - | Skin: -  Mucosa: - | - | + | + | + | 1 (0-1) |
| B6.SJL-H2s C3c/1CyJ  #4 | - | - | - | Skin: -  Mucosa: - | - | + | + | + | 0 (0-1) |
| B6.SJL-H2s C3c/1CyJ  #5 | - | - | n.d. | Skin: -  Mucosa: - | - | + | + | - | 0 (0-2) |
| B6.SJL-H2s C3c/1CyJ  #6 | - | - | n.d. | Skin: -  Mucosa: - | - | + | + | - | 0 (0-1) |
| B6.SJL-H2s C3c/1CyJ  #7 | - | - | n.d. | Skin: -  Mucosa: - | - | + | + | - | 0 (0-1) |
| B6.SJL-H2s C3c/1CyJ  #8 | - | - | n.d. | Skin: -  Mucosa: - | - | + | + | - | 0 (0-2) |
| DBA2/J  #1 | - | - | n.d. | Skin: -  Mucosa: - | - | + | + | - | 0 (0-1) |
| DBA2/J  #2 | - | - | - | Skin: -  Mucosa: - | - | + | + | + | 1 (0-1) |
| DBA2/J  #3 | - | - | n.d. | Skin: -  Mucosa: - | - | + | + | - | 0 (0-2) |
| DBA2/J  #4 | - | - | n.d. | Skin: -  Mucosa: - | - | + | + | - | 0 (0-2) |
| DBA2/J  #5 | - | - | - | Skin: -  Mucosa: - | - | + | + | + | 0 (0-1) |
| DBA2/J  #6 | - | - | - | Skin: -  Mucosa: - | - | + | + | + | 0 (0-1) |
| DBA2/J  #7 | - | - | n.d. | Skin: -  Mucosa: - | - | + | + | - | 0 (0-1) |
| DBA2/J  #8 | - | - | - | Skin: -  Mucosa: - | - | + | + | + | 1 (0-1) |
| SJL/J  #1 | - | - | - | Skin: -  Mucosa: - | - | + | + | + | 0 (0-1) |
| SJL/J  #2 | - | - | - | Skin: -  Mucosa: - | - | + | + | + | 0 (0-1) |
| SJL/J  #3 | - | - | n.d. | Skin: -  Mucosa: - | - | + | + | - | 0 (0-2) |
| SJL/J  #4 | - | - | n.d. | Skin: -  Mucosa: - | - | + | + | - | 0 (0-2) |
| SJL/J  #5 | - | - | n.d. | Skin: -  Mucosa: - | - | + | + | - | 0 (0-1) |
| SJL/J  #6 | - | - | - | Skin: -  Mucosa: - | - | + | + | + | 1 (0-1) |
| SJL/J  #7 | - | - | n.d. | Skin: -  Mucosa: - | - | + | + | - | 1 (0-1) |
| SJL/J  #8 | - | - | - | Skin: -  Mucosa: - | - | + | + | + | 0 (0-1) |
| DBA2/J  #1 + ETA | - | - | - | Skin: -  Mucosa: - | - | + | n.d. | + | 0 (0-1) |
| DBA2/J  #2 +ETA | - | - | - | Skin: -  Mucosa: - | - | + | n.d. | + | 0 (0-2) |
| DBA2/J  #3 +ETA | - | - | - | Skin: -  Mucosa: - | - | + | n.d. | + | 1 (0-3) |
| DBA2/J  #4+ ETA | - | - | - | Skin: -  Mucosa: - | - | + | n.d. | + | 0 (0-1) |
| SJL/J  #1 + ETA | - | - | - | Skin: -  Mucosa: - | - | + | n.d. | + | 0 (0-1) |
| SJL/J  #2 + ETA | - | - | n.d. | Skin: -  Mucosa: - | - | + | n.d. | - | 0 (0-1) |
| SJL/J  #3+ ETA | - | - | - | Skin: -  Mucosa: - | - | + | n.d. | + | 0 (0-2) |
| SJL/J  #4 +ETA | - | - | - | Skin: -  Mucosa: - | - | + | n.d. | + | 1 (0-1) |
| DBA2/J  #1 + hDg1/3 | - | - | - | Skin: -  Mucosa: - | - | + | n.d. | + | 1 (0-1) |
| DBA2/J  #2+ hDg1/3 | - | - | - | Skin: -  Mucosa: - | - | + | n.d. | + | 0 (0-2) |
| DBA2/J  #3+ hDg1/3 | - | - | n.d. | Skin: -  Mucosa: - | - | + | n.d. | - | 1 (0-1) |
| DBA2/J  #4 + hDg1/3 | - | - | - | Skin: -  Mucosa: - | - | + | n.d. | + | 0 (0-1) |
| SJL/J  #1 + hDsg1/3 | - | - | - | Skin: -  Mucosa: - | - | + | n.d. | + | 1 (0-2) |
| SJL/J  #2 + hDsg1/3 | - | - | - | Skin: -  Mucosa: - | - | + | n.d. | + | 0 (0-2) |
| SJL/J  #3 + hDsg1/3 | - | - | - | Skin: -  Mucosa: - | - | + | n.d. | + | 1 (0-1) |
| SJL/J  #4 + hDsg1/3 | - | - | n.d. | Skin: -  Mucosa: - | - | + | n.d. | - | 1 (0-1) |
| SJL/J  #5 + hDsg1/3 | - | - | - | Skin: -  Mucosa: - | - | + | n.d. | + | 0 (0-1) |
| SJL/J  #6 + hDsg1/3 | - | - | - | Skin: -  Mucosa: - | - | + | n.d. | + | 0 (0-1) |

**S2 Table. Result database.**

Mice were weekly weighed and clinically examined for evidence of cutaneous/mucosal lesions to determine primary endpoints. Secondary endpoints were defined by direct immunofluorescence microscopy (DIF) of perilesional biopsies and indirect immunofluorescence microscopy (IIF) (semi-quantitative evaluation of circulating autoantibodies) as well as enzyme-linked immunosorbent assay (ELISA, quantitative evaluation of circulating autoantibodies) of serum samples. At the end of the observation period an oral endoscopy was performed to determine the oral mucosa involvement. Immune staining was performed to detect autoantigen-specific B cells (mDsg3-reactive B/plasma cells) in draining lymph nodes. An adverse events score was performed to sum up all side adverse effects.

**Abbreviation:** DIF, direct immunofluorescence microscopy; ELISA, Enzyme-linked immunosorbent assay; ETA, Exfoliative toxin A; hDg1/3, human Desmoglein 1 and 3; IIF, indirect immunofluorescence microscopy; mDsg3, murine Desmoglein 3; n.d., not done; SD, standard deviation
