# Supplementary material for: Immunization with desmoglein 3 induces non-pathogenic autoantibodies in mice
Source: PLoS One. 2021 Nov 3;16(11):e0259586. doi: 10.1371/journal.pone.0259586 (PMC8565724; doi:10.1371/journal.pone.0259586)
Supplement: S3 Table — The sum of all side adverse effects was added to a total sum. (DOCX) [file pone.0259586.s003.docx]

| **I Body weight** | **value** |
| --- | --- |
| unaffected or rise | 0 |
| Weight reduction <5 % compared to day 0 of the experiment | 1 |
| Weight reduction 5-10 % compared to day 0 of the experiment | 5 |
| Weight reduction 11-20 % compared to day 0 of the experiment | 10 |
| Weight reduction >20 % compared to day 0 of the experiment | 20 |
| **II General condition** |  |
| Coat smooth, shiny, body openings clean, eyes clear, shiny | 0 |
| Fur blunt, disorderly, unkempt body openings, eyes cloudy, increased muscle tone | 1 |
| Dirty coat, sticky or wet body openings, abnormal posture, eyes cloudy, increased muscle tone | 5 |
| Dirty coat, sticky or damp body openings, abnormal posture, eyes cloudy; high muscle tone | 10 |
| Cramps, paralysis (trunk muscles, extremities),wheezing, animal feels cold | 20 |
| **III Spontaneous behavior** |  |
| Normal behavior (sleeping, reaction to blowing and touching, curiosity, social contacts) | 0 |
| Small deviations from the normal behavior | 1 |
| Unusual behavior, impaired motor function or hyperkinetics (noticeable scratching over 2 of 10 min) | 5 |
| Self-isolation, lethargy, pronounced hyperkinetics or behavioral stereotypies, coordination disorders hyperkinetics (noticeable scratching behavior over 5 of 10 min) | 10 |
| Repeated pain sounds when grasping, self-amputation (autoaggression, autotomy) | 20 |
| **IV Clinical findings (measurements of temperature and respiration only if abnormalities are observed under II and III)** |  |
| Temperature, respiration and pulse normal, extremities warm, mucous membranes well supplied with blood | 0 |
| Small deviations from the normal situation | 1 |
| Temperature deviation 1 - 2 °C, respiration and pulse + 30% | 5 |
| Temperature deviation> 2 °C, respiration / pulse +/- 50% | 10 |
| Temperature, respiration and pulse normal, extremities warm, mucous membranes well supplied with blood | 20 |
| **Sum** | **0-80** |

S3 Table. Adverse events score. The sum of all side adverse effects was added to a total sum.
