# Supplementary material for: Immunization with desmoglein 3 induces non-pathogenic autoantibodies in mice
Source: PLoS One. 2021 Nov 3;16(11):e0259586. doi: 10.1371/journal.pone.0259586 (PMC8565724; doi:10.1371/journal.pone.0259586)
Supplement: S4 Table — The mean and standard deviation (SD) of the adverse effects of the experiments is summarized in here. Immunizations did not increase the burden of mice during as the adverse events score is not significantly changed. (DOCX) [file pone.0259586.s004.docx]

| **Immunization** | **Adverse events score (mean ± SD)** |
| --- | --- |
| mDsg3/Titermax |  |
| C57Bl/6J | 0 (1-3) |
| B6.SJL-H2s C3c/1CyJ | 0 (0-2) |
| DBA2/J | 0 (0-2) |
| SJL/J | 0 (0-2) |
| mDsg3/Titermax + ETA |  |
| DBA2/J | 0 (0-3) |
| SJL/J | 0 (0-2) |
| hDsg1/3/Titermax |  |
| DBA2/J | 0,5 (0-2) |
| SJL/J | 0,5 (0,2) |

**S4 Table. Results adverse events score.** The mean and standard deviation (SD) of the adverse effects of the experiments is summarized in here. Immunizations did not increase the burden of mice during as the adverse events score is not significantly changed.
